# Supplementary material for: Correlation between histogram-based DCE-MRI parameters and 18F-FDG PET values in oropharyngeal squamous cell carcinoma: Evaluation in primary tumors and metastatic nodes
Source: PLoS One. 2020 Mar 2;15(3):e0229611. doi: 10.1371/journal.pone.0229611 (PMC7051076; doi:10.1371/journal.pone.0229611)
Supplement: S1 Table — (DOCX) [file pone.0229611.s001.docx]

**S1 Table. Results of Spearman's correlation tests between K^trans^ and ^18^F-FDG-PET parameters in primary tumors (N = 47).**

| *Variables* |  | SUV_max_ | SUV_peak_ | SUV_mean_ | SD | TLG | MTV |
| --- | --- | --- | --- | --- | --- | --- | --- |
| P10 | Rho | ,054 | ,056 | ,051 | ,063 | ,076 | -,018 |
|  | P | ,721 | ,710 | ,735 | ,676 | ,610 | ,904 |
| P25 | Rho | ,107 | ,101 | ,096 | ,094 | ,121 | ,018 |
|  | P | ,476 | ,501 | ,521 | ,528 | ,419 | ,903 |
| P50 | Rho | ,145 | ,144 | ,138 | ,138 | ,121 | -,006 |
|  | P | ,332 | ,336 | ,355 | ,355 | ,420 | ,968 |
| P75 | Rho | ,117 | ,126 | ,124 | ,132 | ,071 | -,039 |
|  | P | ,433 | ,399 | ,408 | ,376 | ,635 | ,795 |
| P90 | Rho | ,120 | ,146 | ,135 | ,143 | ,049 | -,038 |
|  | P | ,422 | ,328 | ,366 | ,337 | ,742 | ,797 |
| skewness | Rho | ,077 | ,042 | ,048 | ,034 | ,251 | ,287 |
|  | P | ,606 | ,778 | ,748 | ,821 | ,089 | ,051 |
| kurtosis | Rho | ,140 | ,098 | ,115 | ,087 | ,280 | ,306^*^ |
|  | P | ,347 | ,513 | ,441 | ,560 | ,057 | ,037 |
| entropy | Rho | ,143 | ,178 | ,159 | ,153 | ,109 | ,024 |
|  | P | ,337 | ,230 | ,286 | ,304 | ,465 | ,874 |

No statistically significant p-value after applying Benjamini-Hockberg correction.
