# Supplementary material for: Correlation between histogram-based DCE-MRI parameters and 18F-FDG PET values in oropharyngeal squamous cell carcinoma: Evaluation in primary tumors and metastatic nodes
Source: PLoS One. 2020 Mar 2;15(3):e0229611. doi: 10.1371/journal.pone.0229611 (PMC7051076; doi:10.1371/journal.pone.0229611)
Supplement: S2 Table — (DOCX) [file pone.0229611.s002.docx]

**S2 Table. Results of Spearman's correlation tests between K_ep_ and ^18^F-FDG-PET parameters in primary tumors (N = 47).**

| *Variables* |  | SUV_max_ | SUV_peak_ | SUV_mean_ | SD | TLG | MTV |
| --- | --- | --- | --- | --- | --- | --- | --- |
| P10 | Rho | -,032 | -,007 | -,007 | ,027 | -,090 | -,209 |
|  | P | ,831 | ,962 | ,965 | ,859 | ,549 | ,159 |
| P25 | Rho | -,052 | -,025 | -,014 | ,037 | -,127 | -,273 |
|  | P | ,728 | ,866 | ,926 | ,804 | ,393 | ,063 |
| P50 | Rho | -,023 | ,045 | ,040 | ,098 | -,082 | -,235 |
|  | P | ,877 | ,764 | ,792 | ,511 | ,582 | ,112 |
| P75 | Rho | -,003 | ,081 | ,068 | ,122 | -,051 | -,185 |
|  | P | ,984 | ,589 | ,650 | ,412 | ,734 | ,213 |
| P90 | Rho | ,032 | ,129 | ,106 | ,157 | -,047 | -,110 |
|  | P | ,833 | ,389 | ,477 | ,293 | ,754 | ,462 |
| skewness | Rho | ,105 | ,112 | ,084 | ,053 | ,270 | ,272 |
|  | P | ,484 | ,453 | ,574 | ,726 | ,066 | ,064 |
| kurtosis | Rho | ,083 | ,099 | ,060 | ,026 | ,289 | ,310 |
|  | P | ,580 | ,507 | ,690 | ,864 | ,049 | ,034 |
| entropy | Rho | ,044 | ,139 | ,118 | ,153 | ,009 | -,076 |
|  | P | ,768 | ,352 | ,430 | ,304 | ,954 | ,610 |

No statistically significant p-value after applying Benjamini-Hockberg correction.
