# Supplementary material for: Correlation between histogram-based DCE-MRI parameters and 18F-FDG PET values in oropharyngeal squamous cell carcinoma: Evaluation in primary tumors and metastatic nodes
Source: PLoS One. 2020 Mar 2;15(3):e0229611. doi: 10.1371/journal.pone.0229611 (PMC7051076; doi:10.1371/journal.pone.0229611)
Supplement: S3 Table — (DOCX) [file pone.0229611.s003.docx]

**S3 Table. Results of Spearman's correlation tests between v_e_ and ^18^F-FDG-PET parameters in primary tumors (N = 47).**

| *Variables* |  | SUV_max_ | SUV_peak_ | SUV_mean_ | SD | TLG | MTV |
| --- | --- | --- | --- | --- | --- | --- | --- |
| P10 | Rho | -,071 | -,143 | -,120 | -,178 | ,069 | ,087 |
|  | P | ,641 | ,348 | ,432 | ,243 | ,650 | ,571 |
| P25 | Rho | ,030 | -,032 | -,021 | -,084 | ,130 | ,168 |
|  | P | ,847 | ,835 | ,891 | ,585 | ,395 | ,270 |
| P50 | Rho | ,093 | ,052 | ,052 | -,015 | ,189 | ,268 |
|  | P | ,543 | ,737 | ,733 | ,921 | ,213 | ,075 |
| P75 | Rho | ,096 | ,038 | ,060 | ,000 | ,188 | ,267 |
|  | P | ,532 | ,804 | ,697 | ,998 | ,217 | ,076 |
| P90 | Rho | ,139 | ,074 | ,112 | ,066 | ,164 | ,238 |
|  | P | ,364 | ,628 | ,463 | ,667 | ,283 | ,116 |
| skewness | Rho | ,040 | -,036 | ,005 | ,064 | -,131 | -,239 |
|  | P | ,794 | ,817 | ,974 | ,677 | ,393 | ,113 |
| kurtosis | Rho | -,202 | -,221 | -,242 | -,188 | -,263 | -,241 |
|  | P | ,182 | ,144 | ,109 | ,216 | ,081 | ,111 |
| entropy | Rho | ,263 | ,240 | ,255 | ,213 | ,265 | ,322 |
|  | P | ,081 | ,113 | ,091 | ,160 | ,079 | ,031 |

No statistically significant p-value after applying Benjamini-Hockberg correction.
