# Supplementary material for: Correlation between histogram-based DCE-MRI parameters and 18F-FDG PET values in oropharyngeal squamous cell carcinoma: Evaluation in primary tumors and metastatic nodes
Source: PLoS One. 2020 Mar 2;15(3):e0229611. doi: 10.1371/journal.pone.0229611 (PMC7051076; doi:10.1371/journal.pone.0229611)
Supplement: S4 Table — (DOCX) [file pone.0229611.s004.docx]

**S4 Table. Results of Spearman's correlation tests between K_ep_ and ^18^F-FDG-PET parameters in lymph nodes (N = 45).**

| *Variables* |  | SUV_max_ | SUV_peak_ | SUV_mean_ | SD | TLG | MTV |
| --- | --- | --- | --- | --- | --- | --- | --- |
| P10 | Rho | -,083 | -,088 | -,086 | -,044 | -,072 | -,058 |
|  | P | ,586 | ,563 | ,572 | ,776 | ,637 | ,706 |
| P25 | Rho | -,312 | -,353 | -,312 | -,253 | -,302 | -,228 |
|  | P | ,037 | ,018 | ,037 | ,093 | ,044 | ,131 |
| P50 | Rho | -,334 | -,376 | -,332 | -,284 | -,339 | -,253 |
|  | P | ,025 | ,011 | ,026 | ,059 | ,023 | ,093 |
| P75 | Rho | -,400 | -,413 | -,404 | -,369 | -,277 | -,147 |
|  | P | ,006 | ,005 | ,006 | ,012 | ,066 | ,337 |
| P90 | Rho | -,387 | -,402 | -,393 | -,369 | -,294 | -,151 |
|  | P | ,009 | ,006 | ,008 | ,013 | ,050 | ,321 |
| skewness | Rho | ,111 | ,106 | ,076 | ,087 | ,115 | ,112 |
|  | P | ,470 | ,487 | ,621 | ,569 | ,451 | ,464 |
| kurtosis | Rho | ,206 | ,199 | ,176 | ,181 | ,184 | ,143 |
|  | P | ,175 | ,191 | ,247 | ,235 | ,228 | ,350 |
| entropy | Rho | -,291 | -,330 | -,298 | -,273 | -,313 | -,224 |
|  | P | ,053 | ,027 | ,047 | ,069 | ,036 | ,139 |

No statistically significant p-value after applying Benjamini-Hockberg correction.
