# Supplementary material for: Correlation between histogram-based DCE-MRI parameters and 18F-FDG PET values in oropharyngeal squamous cell carcinoma: Evaluation in primary tumors and metastatic nodes
Source: PLoS One. 2020 Mar 2;15(3):e0229611. doi: 10.1371/journal.pone.0229611 (PMC7051076; doi:10.1371/journal.pone.0229611)
Supplement: S5 Table — (DOCX) [file pone.0229611.s005.docx]

**S5 Table. Results of Spearman's correlation tests between v_e_ and ^18^F-FDG-PET parameters in lymph nodes (N = 45).**

| *Variables* |  | SUV_max_ | SUV_peak_ | SUV_mean_ | SD | TLG | MTV |
| --- | --- | --- | --- | --- | --- | --- | --- |
| P10 | Rho | ,246 | ,255 | ,251 | ,261 | ,175 | ,016 |
|  | P | ,112 | ,098 | ,104 | ,090 | ,260 | ,919 |
| P25 | Rho | ,182 | ,219 | ,188 | ,194 | ,167 | ,037 |
|  | P | ,242 | ,158 | ,228 | ,213 | ,284 | ,812 |
| P50 | Rho | ,147 | ,188 | ,154 | ,147 | ,117 | ,011 |
|  | P | ,348 | ,226 | ,326 | ,348 | ,455 | ,943 |
| P75 | Rho | ,112 | ,154 | ,115 | ,107 | ,102 | ,019 |
|  | P | ,475 | ,323 | ,462 | ,495 | ,516 | ,904 |
| P90 | Rho | ,125 | ,151 | ,124 | ,116 | ,095 | ,008 |
|  | P | ,425 | ,333 | ,430 | ,460 | ,547 | ,958 |
| skewness | Rho | ,064 | -,005 | ,075 | ,087 | -,110 | -,203 |
|  | P | ,684 | ,975 | ,631 | ,578 | ,481 | ,193 |
| kurtosis | Rho | ,150 | ,080 | ,160 | ,179 | -,079 | -,215 |
|  | P | ,337 | ,609 | ,305 | ,251 | ,614 | ,166 |
| entropy | Rho | ,070 | ,110 | ,087 | ,052 | ,098 | ,056 |
|  | P | ,648 | ,470 | ,570 | ,734 | ,523 | ,715 |

No statistically significant p-value after applying Benjamini-Hockberg correction.

Study database, including patient/tumor characteristics and quantitative analyses from FDG-PET and DCE-MRI imaging for both primary tumors and lymph nodes.
